# Case Report: Acute Spinal Cord Myelopathy in Patients With COVID-19

**DOI:** 10.3389/fneur.2020.610648

**Published:** 2020-12-22

**Authors:** Eman M. Khedr, Ahmed A. Karim, Radwa K. Soliman

**Affiliations:** ^1^Department of Neuropsychiatry, Assiut University Hospitals, Assiut, Egypt; ^2^Department of Psychiatry and Psychotherapy, University of Tübingen, Tübingen, Germany; ^3^Department of Radiology, Assiut University Hospitals, Assiut, Egypt

**Keywords:** case report, COVID-19, spinal cord myelopathy, anterior spinal artery occlusion, transverse myelitis, anterior spinal artery infarct, magnetic resonance image (MRI), SARC V2

## Abstract

COVID-19 is typically associated with fever and severe respiratory symptoms including dry cough and dyspnea. However, COVID-19 may also affect both central and peripheral nervous systems. To date, the incidence rate of spinal cord involvement in COVID-19 is not known and the pathogenesis is still not fully understood. We report here two female patients admitted to Assiut University Hospitals/Egypt during the period from first of July to August 10, 2020. Both presented with a positive SARS-CoV-2 polymerase chain reaction (PCR) nasopharyngeal swab, elevated serum d-dimer and ferritin levels, and bilateral ground glass appearance in a CT chest scan. The first was a 60-year-old female with acute onset of flaccid paraplegia 10 days after flu-like symptoms, in whom MRI revealed transverse myelitis. The second was a 21-year-old female with symptoms of acute quadriplegia, fever, headache, and anosmia in whom an MRI scan revealed long cervico-thoracic myelopathy. Anterior spinal artery occlusion and possibly transverse myelitis were considered as differential diagnosis of long segment myelopathy.

## Introduction

The pandemic of the coronavirus disease 2019 (COVID-19) is spreading rapidly across the world. COVID-19 is typically associated with fever and severe respiratory symptoms including dry cough and dyspnea. Interestingly, there have been several reports of cases with neurological symptoms (1) affecting both central and peripheral nervous systems. The most common serious central nervous system (CNS) complications are cerebrovascular diseases (1). However, spinal cord involvement seems to be rare in COVID-19. To our knowledge, only a few case reports have been published (2–5).

Here, we discuss two case reports of spinal cord myelopathy following acute COVID-19 pneumonia: the first with acute flaccid paraplegia caused by transverse myelitis (post-infection), and the second with acute quadriplegia and cervicothoracic myelopathy secondary to anterior spinal artery occlusion or possibly transverse myelitis.

## Case Presentation 1

A 60-year-old female came to the hospital with a 3-day history of high-grade fever and dry cough followed by weakness and numbness of both lower limbs (walking only with support) with girdle-like pain at the mid-thoracic level. Two days later, the weakness had progressed to complete lower limb paralysis with loss of sensation below the T4 level, retention of urine and fecal incontinence. The patient's previous medical history was innocuous apart from hypothyroidism. She is presently treated with Levothroxine (Eltroxine 100 mg once daily on an empty stomach). There was no personal or family history of previous neurological disorder. She had received no vaccinations in previous months.

On examination: Mental state was normal and speech and cranial nerves were unaffected. There was complete paralysis of both lower limbs, with hypotonia and hyporeflexia and a positive Babinski sign bilaterally. Sensation to light touch was diminished below T4 with loss of pricking pain, temperature sensation and vibration (tested by a tuning fork).

Laboratory tests: SARS-CoV-2 polymerase chain reaction (PCR) was positive in a nasopharyngeal swab, with elevated serum d-dimer (8 mg/L) and ferritin (350 ng/mL) (reference values: d-dimer up to 0.55 mg/L and ferritin 10–291 ng/mL). C-reactive protein (CRP) and erythrocytic sedimentation rate (ESR) 1st and 2nd hour were also elevated (120 mg/dL, 90 and 160 for the 1st and 2nd hour, respectively) (reference value; CRP up to 1 mg/dL, ESR; 1st and 2nd hour 5–10 mm/H). WBCs 7.9 × 10^9^/μL, RBC 4.18 × 10^6^/ μL, hemoglobin 10.70 g/dL, platelet 188 × 10^3^/μL, neutrophils 80.9% (high), lymphocytes 15% (low), eosinophil 0.70 (low), basophil 0% and monocyte 2% (reference values: WBCs 4–10 × 10^3^/μL, hemoglobin 12–15 g/dL, platelets 140–450 × 10^3^/μL, relative neutrophil 40–75%, relative lymphocytes 20–45%, relative monocytes 2–10%), eosinophils (2–6%), and basophils (0–1%). All electrolytes were normal (Na^+^, K^+^, Mg^2+^, and Ca^2+^). Liver and renal functions were normal.

**Figure 1:**
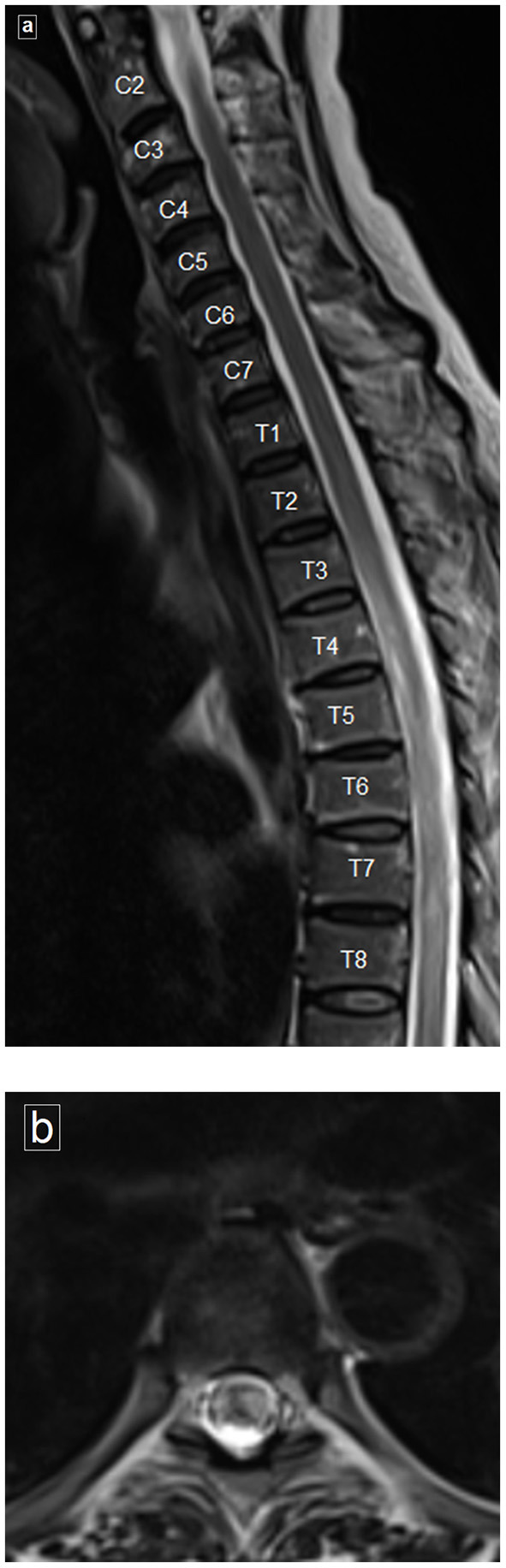
Magnetic resonance imaging (MRI) of spine. **(a)** Sagittal T2-weighted image of the cervical and dorsal spine reveals poorly delineated long segment of hyperintense signal, clearly evident from T4 to T8. **(b)** Axial T2-weighted image showing the hyperintense signal involving more than two thirds of the cross-sectional area of the cord.

There was a bilateral ground glass appearance on chest CT. A sagittal T2-weighted MRI image of the cervical and dorsal spine (Figures 1a,b) revealed a poorly delineated long segment of hyperintense signal extending from T4 to T8 involving the central region and occupying more than two-thirds of the cross-sectional area of the cord, consistent with transverse myelitis. An axial T2-weighted image shows the central hyperintense signal involving the cord.

The patient was treated with methylprednisolone 1 g IV for 5 days followed by slow oral prednisone tapering for 2 weeks, which resulted in no improvement. Randomized trials have validated the use of plasma exchange in severe cases that are unresponsive to pulsed corticosteroid administration (6, 7); the patient received five sessions of plasma-pharesis (one session every 2 days), but with no improvement. After the fourth session of plasma-pharesis, she developed right ilio-femoral deep venous thrombosis (DVT). She received low molecular heparin 0.6 IU subcutaneously every 6 h for 5 days with improvement of DVT but the lower limb paralysis was not improved. A few days later, she had an attack of severe dyspnea and cyanosis with pulmonary embolism as confirmed by CT scan of the chest. She was ventilated but died a few hours later.

## Case Presentation 2

A 21-year-old otherwise healthy female came to our clinic with a 10-day history of high-grade fever, dry cough, and anosmia followed by acute onset (taking few hours) flaccid paralysis and numbness of both lower limbs with difficulty in voiding. One day later, she developed weakness and numbness of both upper limbs with retention of urine and fecal incontinence. Autonomic symptoms included fluctuations of heart rate with frequent attacks of palpitation, and dizziness and fainting upon sitting from recumbent position. She had received no vaccinations in previous months, and there was no history of a previous similar attack. On general examination, heart was free with normal blood pressure and symmetry of both arms. On neurological examination, she had quadriplegia with greater involvement of the lower limbs, areflexia, and a bilateral Babinski sign. Pain and temperature sensation was absent up to the C4 level with preserved touch, vibration, and joint position sense.

Laboratory tests: SARS-CoV-2 PCR was positive in a nasopharyngeal swab, and serum d-dimer (4.6 mg/L) and ferritin (502 ng/mL) were elevated. Blood counts were as follows: neutrophils 85.7% (high), lymphocytes 17% (low), eosinophil 0.50 (low), basophil 0% and monocyte 3%, WBCs 6.7 × 10^3^/μL, RBC 4.9 × 10^6^/μL, hemoglobin 10.8 g/dL, platelet 333 × 10^3^/μL. Electrolytes were normal (Na^+^, K^+^, Mg^2+^, Ca^2+^, and PO_4_). Elevated ESR 1st hour 100 and 150 for 2nd hour, CRP (111 mg/dL), and lactic dehydrogenase (LDH) (356 U/L) were also recorded (reference value; LDH 100–190 U/L). Coagulation profile was normal (prothrombin time 12.8 s, concentration 96%, and INR 1.03) (normal value of coagulation profile prothrombin time was 11–14 s, prothrombin concentrate was 70–130%, PTT was 26–40 s, and INR was 0.5–1.07).

Visual and brain stem auditory evoked potentials were normal. Chest CT showed a bilateral ground glass appearance with no evidence of aortic dissection. Electrocardiography was normal.

**Figure 2:**
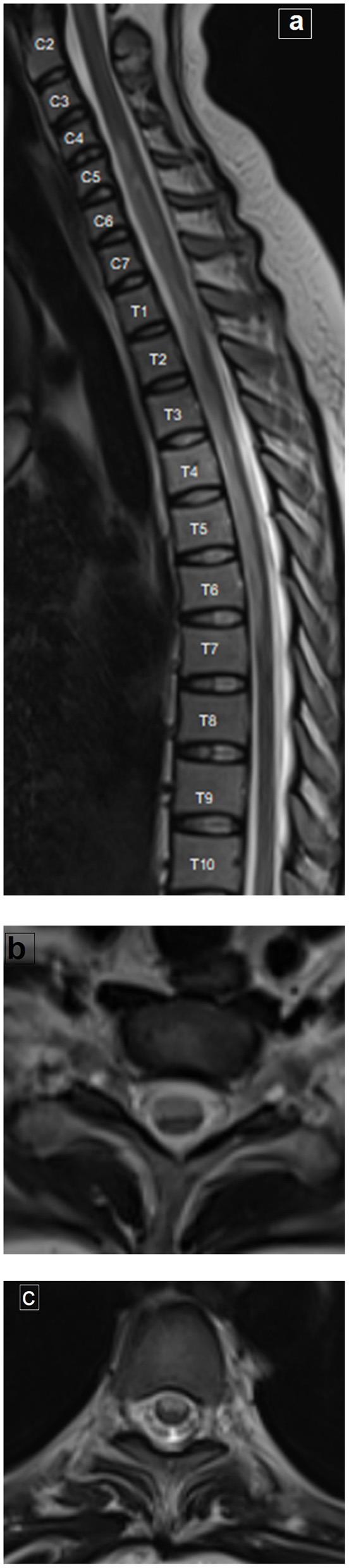
MRI of the spine. **(a)** Sagittal T2-weighted image of the whole spine shows extensive long hyperintense signal extending from C5 to T7, associated with mild cord swelling. **(b, c)** Axial T2-weighted images at the cervical and dorsal levels, respectively, showing the hyperintense signal occupying the anterior two thirds of the cord, bilaterally (with involvement of both the gray and white matter).

MRI examination of the spine revealed an extensive hyperintense signal on T2-weighted imaging, extending from C5 to T7, and occupying the anterior two thirds of the cord with involvement of both gray and white matter. This was associated with mild swelling of the cord (Figures 2a–c). The possibility of anterior spinal artery occlusion was considered given the clinical presentation, laboratory, and MRI findings. A CT of the chest showed no evidence of aortic dissection with normal heart and symmetrical blood pressure in both arms. Thus, we think it unlikely that the lesion was the result of aortic dissection-related occlusion of multiple supplying arteries. Given her age, multiple sclerosis was considered as a possible diagnosis. However, the long segment of cord involvement, together with the normal evoked potentials and no positive MRI findings in the brain, excluded multiple sclerosis. The possibility of transverse myelitis was also considered as a differential diagnosis of long segment myelopathy; however, the complete preservation of deep sensation on clinical examination could support the diagnosis of anterior spinal artery occlusion.

The patient was treated with methylprednisolone 1 g IV for 5 days but no improvement. Corticosteroid was, therefore, stopped gradually over the next 10 days and the patient was treated with Rivaroxaban (20 mg/day) for 30 days with short wave therapy and passive exercise without improvement. Finally, she received intravenous immunoglobulin (IVIG) 0.4 g/kg/day for 5 consecutive days and was followed up for another month. There was mild improvement in upper limb strength while the lower limbs were still flaccid.

## Discussion

Human coronaviruses can be neuroinvasive and neurotropic (7, 8). In particular, SARS-CoV appears to cause a variety of both CNS and PNS disorders such as cerebrovascular stroke, encephalitis, Guillain–Barre Syndrome, and isolated cranial nerve palsy. Although the pathophysiological mechanisms are unclear, there are two likely possibilities: hematogenous dissemination (9) and neuronal retrograde dissemination (10). The first requires the presence of virus in the blood, where it can infect the endothelium. In contrast, neuronal retrograde dissemination occurs when the virus infects sensory or motor nerve endings and is transported retrogradely or anterogradely into the CNS (11, 12). Transverse myelitis is a rare complication of COVID-19 and it is still unsettled whether the myelitis is a direct result of viral infection or an autoimmune sequelae.

Our first case involved dorsal transverse myelitis occurring shortly after COVID-19 infection. In transverse myelitis, the lesion usually involves the central part of the cord, occupying more than two thirds of its cross section and extends longitudinally over more than one segment (13). Since no other causes of myelitis could be identified and since inflammatory markers (d-dimer, ferritin level, CRP, and ESR) were very high, we postulated that this was due to a post-infectious secondary immunogenic overreaction. Others have previously shown that human coronaviruses like SARS can directly infect the central nervous system (14), and it has been suggested that this could provoke systemic cytokine production, which might contribute to the pathophysiology of COVID-19 (15).

To date, four reports of similar cases have been published in the literature, all of which link COVID-19 to acute myelitis as a neurological complication. The first was in Wuhan, China, where Zhao et al. (16) described a 66-year-old patient who developed acute flaccid paraplegia and urinary incontinence. They postulated focal myelitis but without MRI imaging or serological confirmation. The second case was in Boston where a 28-year-old female with a history of hypothyroidism and treatment with levothyroxine developed symptoms of productive cough and low-grade temperature followed by acute myelitis 7 days later (4). In the third case, Munz et al. (3) reported a 60-year-old patient with acute onset moderate spastic paraparesis with retention of urine. An MRI of the spine revealed a T2 signal hyperintensity at Th 9 level suggestive of acute transverse myelitis. AlKetbi et al. (2) reported the fourth case of acute myelitis in a 32-year-old male COVID-19-positive patient. Two days after presenting with flu-like symptoms, he experienced a sudden-onset paraplegia with urinary retention. A spinal MRI revealed a large volume of hyperintense signal involving predominantly the gray matter in the cervical, dorsal, and lumbar regions.

Our second case is the first report of a young female, initially diagnosed as transverse myelitis. Yet, as she presented clinically with characteristic features of acute paralysis of four limbs with loss of pain and temperature together with preserved dorsal column function and MRI findings of bilateral involvement of the anterior 2/3 of spinal cord, we considered the possibility of anterior spinal artery occlusion. Diffusion MRI would be helpful to confirm such diagnosis at early onset. Unfortunately, an MRI scan was only available at a later stage for this patient. Although the extensive longitudinal involvement along the cord (as shown in our case) is most frequently associated with large vessel dissection, other causes of thromboembolism and coagulopathy also have been shown to be associated with long segment involvement (17).

As our patient is young with no vascular risk factors, and no evidence of aortic or vertebral dissection but had high levels of inflammatory markers (such as CRP, ESR, and ferritin) and markers of coagulation such as d-dimer, we consider that her condition was most probably related to vasculopathy as well as hypercoagulopathy. In line with this suggestion, Beyrouti and co-workers reported six severely affected patients who had large cerebral infarcts and elevated d-dimer levels (≥1,000 μg/L) consistent with a coagulopathy (18).

The fact that young people with COVID-19 have an unexpectedly high frequency of ischemic stroke but few risk factors may mean that other causes peculiar to COVID-19 are at play. One possibility is that viral invasion of the vascular endothelium (endotheliitis) contributes to vascular ischemia of the spinal cord.

The diagnosis of MS clinically isolated syndrome can be suspected at this age; however, the absence of clinical and MRI finding of dissemination in space and the involvement of multiple segments with the long hyperintense signal as appeared on MRI spine negate this possibility.

### Limitations

The absence of diffusion imaging in the second case is a major limitation since this would help to confirm the diagnosis of spinal artery infarction. However, MRI was only available at a later stage.

## Data Availability Statement

The raw data supporting the conclusions of this article will be made available by the authors, without undue reservation.

## Ethics Statement

The studies involving human participants were reviewed and approved by Faculty of Medicine, Medical Ethics Committee. Assiut University IBR no 17300470. The patients/participants provided their written informed consent to participate in this study. Written informed consent was obtained from the individual(s) for the publication of any potentially identifiable images or data included in this article.

## Author Contributions

EK performed neurological examination and wrote the first draft of the article and is responsible for the overall content. RS has evaluated the radiological images and contributed mainly to the discussion of the cases. AK and the two authors contributed significantly to the discussion of the patient and revision of the first draft. All authors approved the final article and assured all the questions regarding the accuracy of the article.

## Conflict of Interest

The authors declare that the research was conducted in the absence of any commercial or financial relationships that could be construed as a potential conflict of interest.
